# Consensual Negotiation-Based Decision Making for Connected Appliances in Smart Home Management Systems

**DOI:** 10.3390/s18072206

**Published:** 2018-07-09

**Authors:** Khac-Hoai Nam Bui, Jason J. Jung, David Camacho

**Affiliations:** 1 Department of Computer Engineering, Chung-Ang University, Seoul 156-756, Korea; hoainam.bk2012@gmail.com; 2 Department of Computer Science, Universidad Autônoma de Madrid, 28049 Madrid, Spain; david.camacho@uam.es

**Keywords:** Internet of Agent, decentralized system, consensual negotiation, smart decision-making, smart home energy management system, load balancing

## Abstract

Recently, the concept of Internet of Agent has been introduced as a potential technology that pushes intelligence, data processing, analytics and communication capabilities down to the point where the data originates. In this paper, we introduce a novel approach for a Decentralized Home Energy Management System by applying the Internet of Agent concept. In particular, we first present an Internet of Agent framework in terms of sensing, communicating and collaborating among connected appliances. Then, the decentralized management based on consensual negotiation mechanism with several intelligent techniques are proposed for dynamic scheduling connected appliance. Specifically, by applying the Internet of Agent framework, connected appliances are regarded as smart agents that are able to make individual decisions by reaching agreement over the exchange of operations on competitive resources. Furthermore, in this study, the load balancing problem in which load shifting is able to reduce the electricity demand during peak hours is taken into account in order to emphasize the effectiveness of our approach. For the experiment, we develop a simulation of smart home environment to evaluate our approach using NetLogo, a tool which provides real-time analysis in the modeling and simulation domain of complex systems.

## 1. Introduction

The evolution of Internet of Things (IoT) enables manually controlled electrical and electronic devices that can be controlled automatically. Technically, Artificial Intelligence (AI) with many techniques, such as Multi-Agent System (MAS), Decision Making (DM), Fuzzy Systems (FS) and Genetic Algorithms (GA), has been adopted in order to enable the intelligent automation for connected objects in IoT systems. However, the high-processing computing problem becomes a challenge that should be considered to implement those approaches in the future of an IoT system which requires faster decision-making processes [1].

**Figure 1:**
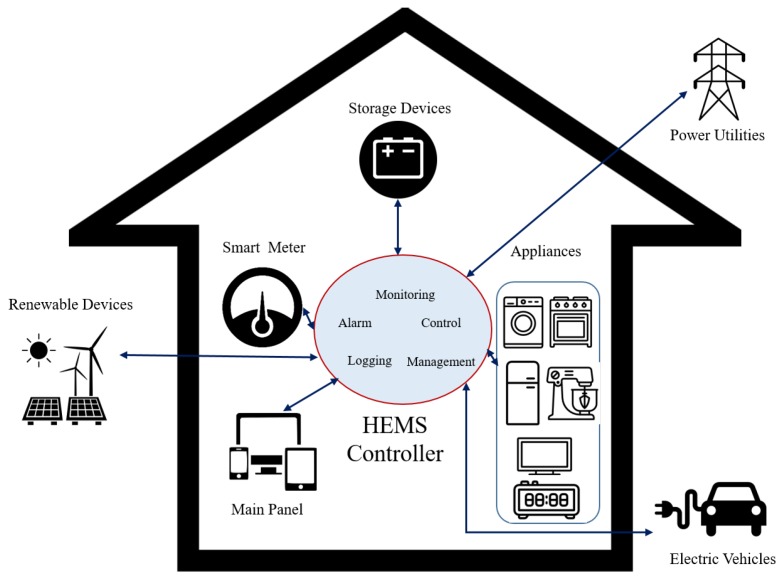
Overall architecture of the Home Energy Management System (HEMS).

In the application of IoT for smart home management, recent advancements on information and communication technologies (e.g., advanced metering infrastructure, smart sensor technologies, bidirectional communication, smart home appliances, home area network, home energy storage system and so on) have been introduced as the promising technologies to provide a technical foundation and infrastructure for a smart house with a home energy management system (HEMS) [2]. Figure 1 shows an overall architecture of HEMS. Particularly, with the development of a home communication network, the HEMS controller is able to provide monitoring modules and control functionalities with real-time electricity consumption data from connected appliances. In addition, the smart meter [3] receives a demand response signal from power utilities as the input data for smart HEMS and monitors the consumer energy usage in order to maintain a balanced load. In this regard, optimization of the home appliance scheduling can be implemented for the residential demand response. Moreover, with the rapid development of sustainable energy technologies and the increasing demand for low-emission generations, the utilization of renewable energy devices such as solar, wind, biomass, and geothermal energy becomes a promising solution for improving the demand response energy consumption of smart HEMS [4].

Based on the advanced technologies of HEMS architecture, there have been many works that focus on energy scheduling strategies in smart house [5,6,7,8]. Most of the existing works take into account centralized management with the evolutionary algorithm to handle either single or multiple objective optimization. However, those systems have to deal with flexibility and scalability problems for the following reasons:

- Smart homes include different types of appliances with different properties and requirements. For instance, in the case of non-scheduled appliances such as lights, computers, televisions and so on, the comfort level of users is quite sensitive and their usage should not be delayed, whenever users require them to be used. In this regard, user satisfaction becomes a challenge that needs to be taken into account. Moreover, the state-of-the-art approaches for smart HEMS focus on centralized management by applying optimizing algorithms such as GA or Particle Swarm Optimization (PSO), which have to face hard computational problems, especially in the case of a high density of connected appliances.
- With the rapid development of IoT, a massive number of electrical appliances will be connected in the near future. In this regard, current approaches with centralized control model (e.g., HEMS controller) have to face the scalability problem in complex systems.
- Furthermore, the power utilization of renewable energy sources keep changing depending on the ambient environment (e.g., time, weather, geography location and so on). Thereby, real-time analytics with self-adaptive management is required for the energy monitoring and dynamic scheduling of home appliances.

Among approaches for smart HEMS, load balancing is introduced as an essential key for energy optimization of modern smart homes in which load shifting is able to reduce the electricity demand during peak hours [9,10]. In particular, time varying pricing models (e.g., Critical Peak Pricing (CPP), Time of Use (ToU) pricing and so on) are introduced by the service provider and can be adopted by the consumer. In this regard, the dynamic scheduling appliances algorithm can support both the service provider and the consumer by shifting their load from peak hours into off-peak hours. Therefore, in this study, we focus on the load balancing problem by proposing a new approach for smart HEMS. Specifically, the proposed approach is a decentralized algorithm and conducted autonomously by intelligent agents, which is able to overcome the aforementioned problems in current works. Accordingly, the motivations and contributions of this paper are defined as follows:

### 1.1. Motivation

For the purpose of participating in electricity saving and demand response in the load balancing problem, HEMS should be more flexible in managing and controlling smart home appliances, renewable energy resources, and home energy storage systems. Moreover, active control services, including real-time information on the amount of energy consumption and the pricing of energy in smart homes, can be provided to consumers on the basis of HEMS in terms of automatic and adaptive systems.

Based on advanced technologies on edge analytics which move the intelligence into the source of network, the study on the decentralized approach among connected objects becomes a research trend for the future of IoT development [11]. Furthermore, by applying a negotiation approach among connected objects, which is expected to be an important issue for enabling intelligence into IoT devices, the system is able to provide automatically self-adaptive decision for multiple objectives energy consumption and comfort level indicator.

### 1.2. Contributions and Organization

The main contributions of this paper include:

- We first present a sustainable framework for HEMS in terms of communicating and collaborating among connected objects in a connected environment in an IoT system.
- Based on the proposed framework, we propose a new algorithm for dynamic scheduling connected appliances by applying negotiation, which is a powerful approach for solving the conflict problem in a complex system. In particular, individual decision-making based on negotiation is considered as a potential approach for pushing intelligence in connected objects, which is the core goal for the future of IoT development.
- Moreover, we design and simulate a smart home environment for evaluating the capabilities of proposed approach by using *Netlogo*, a tool for real-time analysis, an important element for adaptive systems in HEMS.

The rest of this paper is organized as follows: in Section 2, we present a brief introduction about the background and literature review for Smart HEMS. In Section 3, we present an IoA framework for modeling the proposed system based on recent advanced technologies in the smart home. Subsequently, the problem formulation and the concept of consensual negotiation for distributed smart HEMS are indicated in Section 4. A dynamic scheduling approach based on a consensual negotiation approach among connected appliances is presented in Section 5. In Section 6, we develop the experiment for home environment using Netlogo simulator in order to evaluate our proposed approach. Section 7 presents the conclusions and outlines future work related to this study.

## 2. Background and Literature Review

### 2.1. Connectivity Infrastructure for the Home-IoT Ecosystem

Under the IoT paradigm, recent advancements on information and communication technologies, such as advanced metering infrastructure (AMI), smart sensor technologies, bidirectional communication, smart home appliances, home area network, home energy storage system, and so on, have enabled reliable two-way communications between power utilities and home consumers [12]. In this regard, the system is able to not only optimize the utilization status of home appliances, but also manage services for distributed energy resources. The in-home infrastructure of a connected home in terms of a smart energy management system includes smart meters, a communication and networking system, and connected devices [2]. Thereby, HEMS can access, monitor, control, and optimize the performance of various connected objects such as Distributed Energy Resources (DER), Electric Vehicles (EV), and household appliances.

**Figure 2:**
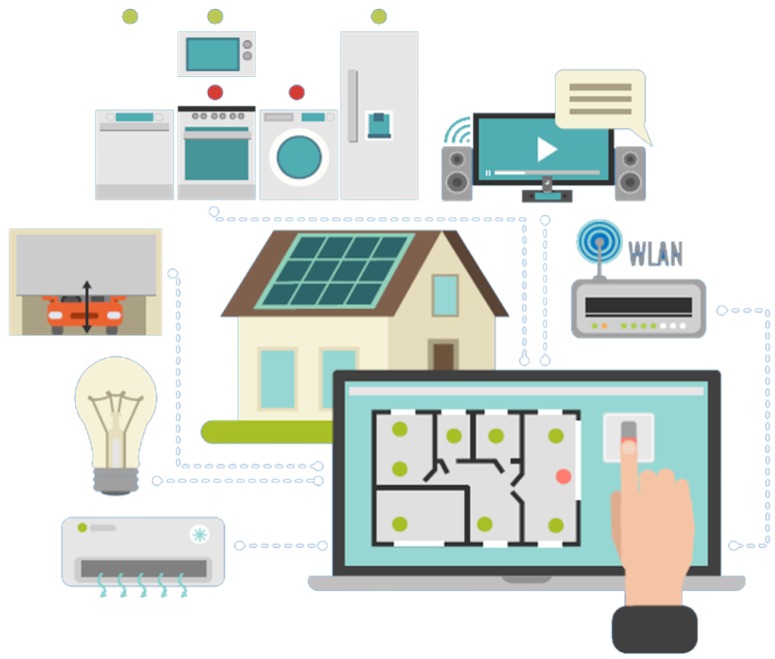
Connected appliance-based home automation.

Consequently, the concept of Home Automation is no longer a futuristic vision [13]. Indeed, increased network bandwidth, round the clock available connectivity, and falling prices of sensor and wireless technologies have enabled connected objects in the home to communicate and collaborate with each other and have made consumers more restless to experience the comfort of managing a smart home (Figure 2). In this paper, we focus on study for a smart home where the advanced technologies enable not only automation, but also the adaptation for smart energy management by applying smart decision-making techniques for connected appliances [13].

### 2.2. Agent Technologies for Negotiation Model-Based Smart Decision-Making

**Figure 3:**
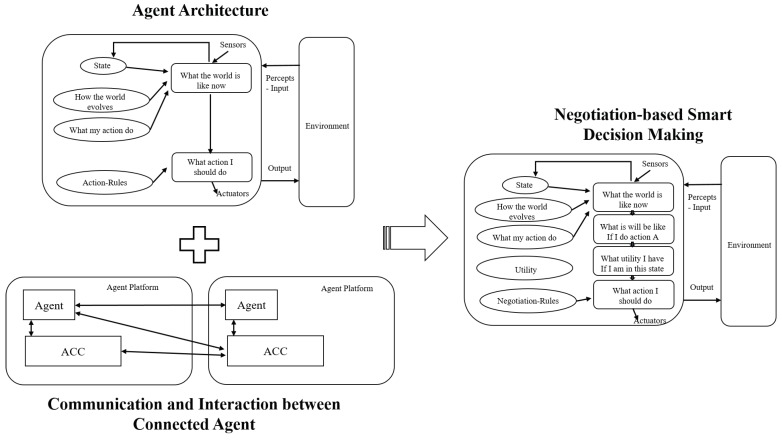
The evolution of intelligent agents.

In the smart application setting based on MAS, agents are represented by hardware of software for learning the preferred environmental conditions, habits, states, and situations of users and subsequently taking appropriate actions [14]. In the literature, MAS consists of four agent groups, which are control and monitoring agents (CMA), Information Agents (IA), Application Agents (AA), and Management and Optimization Agents (MOA). Recently, with the development of IoT, the term IoA [15] has been introduced based on the combination of IoT and MAS, which enables IoT to adapt with intelligent architectures. In particular, the development of IoT devices requires decentralization and distribution in order to increase flexibility, agility, and dependability. In this regard, IoA makes it possible to increase the autonomy and flexibility in the industrial environment, which allows for an increase in terms of integration and interoperability of applications and systems. For instance, Figure 3 shows the evolution of intelligent agents for smart decision-making based on negotiation within the World Wide Web. Specifically, based on advanced technologies in IoT environment, agents are regarded as smart objects that are able to communicate and interact with each other.

Thereby, there have been many studies which have focused on individualized intelligent decision-making and automated negotiation among agents (connected objects) with which to provide smart applications [16,17,18,19]. Normally, the challenge of automated negotiation is to design decision-making approaches for allocating resources (negotiation issue) by negotiating (negotiation process) among intelligent agents following a rule-based system (negotiation protocol) of the smart application. Particularly, we can classify the negotiation approach into two models such as theoretical and computational models, which are defined as follows [20]:

- Theoretical models for describing, specifying, and reasoning about the key features of negotiating agents.
- Computational models for specifying the key data structures of negotiating agents and the processes operating on these structures.

Technically, computational models (e.g., game theoretic approach, heuristic approach, and so on) are used successfully in a wide variety of real-world domains compared with theoretical models. Specifically, in the concept of theoretical models, the assumptions fail in most realistic environments due to the limited processing and communication capabilities of existing systems. However, as we mention, with the successful development of network connectivity (e.g., Sensor, Wireless Network and so on), this problem can be solved in the very near future. In this regard, in this study, we apply consensual negotiation based on a theoretical model for decentralized energy management system among connected appliances instead of a computational model that has trouble dealing with complex systems.

## 3. Internet of Agent Framework among Connected Appliances for Smart HEMS

**Figure 4:**
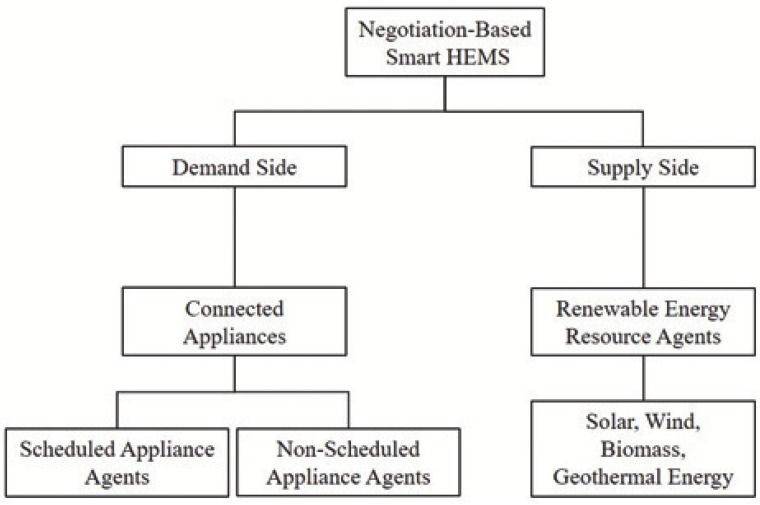
Agent architecture for smart home energy management.

**Figure 5:**
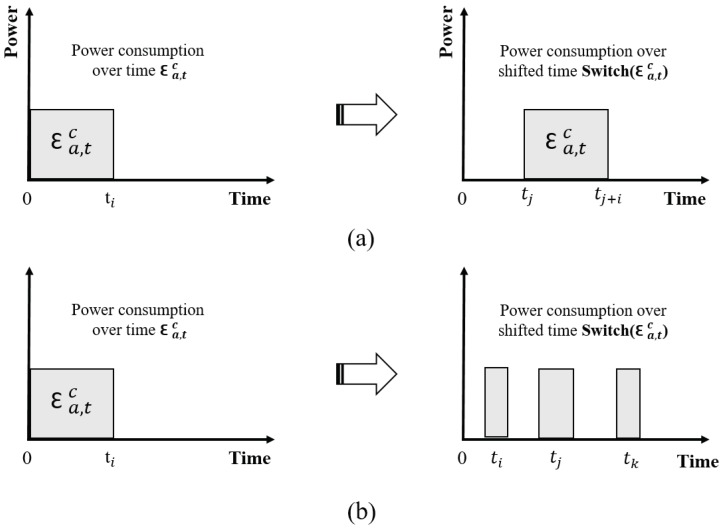
(**a**) generic load shift model; (**b**) flexible load shift model.

In order to propose a decentralized approach for smart HEMS, we first present a sustainable framework in terms of communicating and collaborating among connected appliances. Specifically, based on the concept of HEMS, there are two major parts of functionalities, which are demand side management and supply side management (Figure 4). Demand side is an important function of an energy management system; it is used to reduce electricity efficiency for the end-users by increasing electricity demands during off-peak hours instead of peak hours, which is expensive. On the other hand, supply side includes power supplies (e.g., electric grid, solar panels, micro wind turbine and so on) to provide energy that use to run electric appliances. In smart homes, based on characteristics and requirements, connected appliances can be divided into three categories:

- Non-scheduled Appliance ($T_{1}$): the appliances which rely on manual control to complete a task and need to operate when customers are home such as lights, ovens, and battery chargers. For the comfort level of customers, the usage of non-scheduled appliances should not be delayed.
- Scheduled Appliance ($T_{2}$): the appliances that can complete a task without any manual control, such as washing machines, dryers, electric cars, water heater and so on. Additionally, scheduled appliances are divided into two types of load shift models (LSM): generic load (GL) and flexible load (FL) shift models (Figure 5).
- Supply Appliance $T_{3}$: the appliances which provide energy for running appliances such as solar panels, and wind turbines.

Regarding the objective of this study, we take into consideration real-time dynamic scheduling appliances ($T_{2}$) in order to meet with the load balancing problem in smart homes based on the status of non-schedule appliances ($T_{1}$) and ($T_{3}$). Normally, there are two main assumptions in load balancing which include (i) the power consumption load for each appliance operation and (ii) the demand response module provides necessary information on energy prices and power limits [21]. As we mention above, in the IoT environment, the development of IoA framework for energy management in smart homes allows for implementing the system in terms of automatically and adaptively by communicating and interacting among connected appliances. Particularly, in the connected environment, each connected appliance is regarded as an intelligent agent that can coordinate and collaborate with each other over the Internet. Generally, the components of an intelligent agent in a connected environment can be defined as follows:

**Definition** **1** **(IoT-based** **Connected** **Appliance).** *A connected appliance (agent) consists of several cooperating units that can be represented by 4-tuples as follows:*

A=〈I,P,K,E〉,

*where*

**Figure 6:**
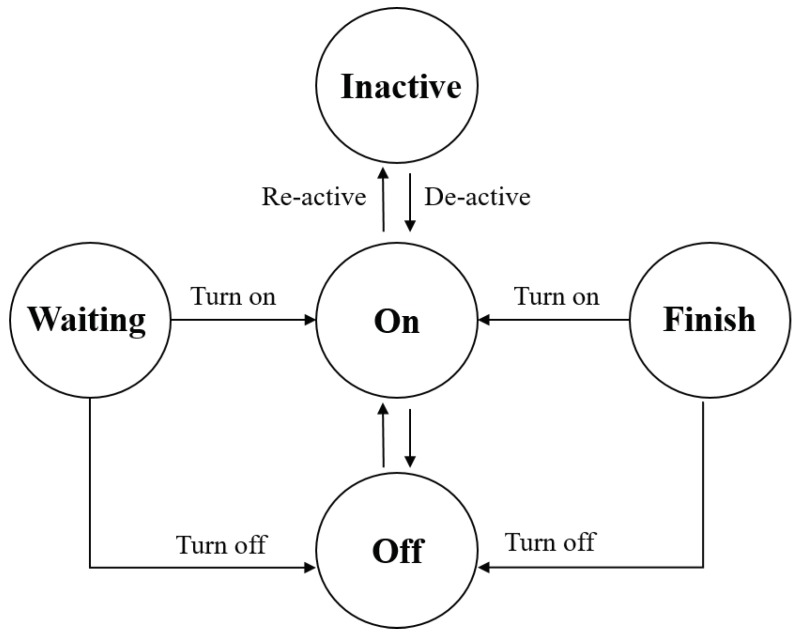
Status actions of connected appliances.

- I is the identification of agent. Technically, each agent has their own id number.
- P is the set of relay ports (interface) which includes the set of agent input and output.
- K: is the Knowledge base which perceives its environment through sensors (Figure 3).
- *E: is state engine which specifies behaviors of agent. The State Engine E can be represented by a tuple as follows:*
  E=〈A,M,U,S〉
  *where*
  - A is action set. The action represents the possible activities of the agent which includes the starting time $\Phi^{s}$ and end time $\Phi^{e}$. For instance, Figure 6 depicts the list of status (actions) of appliances.
  - M is the set of message exchanges including incoming and outgoing messages.
  - U: is the electric utility function when an agent implements an action a∈A.
  - S: is the set of states of the agent. State of agent can be passive or active.

Regarding the communication protocols, connected appliances communicate and collaborate with each other via message protocol in real time [13]. Basically, there are some standard languages for agent communications such as Foundation for Intelligent Physical Agents (FIPA) or Knowledge Query and Manipulation Language (KQML) [22]. However, to simplify the problem in a smart home management system, in this paper, we define a new performative for exchange messages among connected appliances following the protocol of message exchange among intelligent agents. The message types of connected appliances are defined as follows:

**Definition** **2** **(Message** **Types).** *There are three types of messages that connected appliances communicate to each other which include:*

- REQUEST: When a connected appliance requests a turn on, it broadcasts a request message to use energy.
- REPLY: When a connected appliance receives a REQUEST message, it may respond with a REPLY message or add the REQUEST message in its pending list.
- RELEASE: When connected appliances complete their operation, it broadcasts REPLY messages to all requests in its pending list.

Note that, in this study, we assume that there is no message loss during transmissions among connected appliances. Particularly, some transmission problems (e.g., message loss, security and so on) are not the focus of our work in this research. In fact, some mechanisms such as timeout-based retransmissions [23] can be used for more reliable communication.

## 4. Problem Formulation

### 4.1. Energy Consumption Formulation

Since the objective of this paper is dynamic scheduling for connected appliances in smart HEMS, the formulations of energy consumption in the smart home are presented. In particular, considering a home with a set of appliances N$\left\{a_{1},a_{2}\ldots a_{n}\right\}$ where $\left\{a_{1},a_{2}\ldots a_{n}\right\}$ represents each appliance over time horizon T. The total energy consumption $E^{c}$ of appliances in a certain time T (e.g., hour, day, week and so on) can be mathematically represented as:

(1) $$E_{T}^{c}=\sum_{t=1}^{T}\left(\sum_{i=1}^{N}E_{a_{i},t}^{c}\right),$$

where $E_{a_{i},t}^{c}$ denotes the energy consumption of the appliance $a_{i}$ in a time slot *t* that can be calculated as follows:

(2) $$E_{a_{i},t}^{c}=P_{a_{i}}\times \gamma_{a_{i}}^{t},$$

where $P_{a_{i}}$ denotes the power rating of appliance $a_{i}$ and $\gamma_{a_{i}}^{t}$ ∈ [0,1] is the operation state (Figure 6) of appliances in time slot *t*. Note that, in this paper, we assume that the power rating of appliances is not changed following time operation. Table 1 shows a list of appliances that we take into account for simulation in smart home systems [24,25].

Furthermore, regarding the renewable energy resources, $E^{p}$ is the available energy that is produced by supplied devices. Hence, the power surplus at a time slot, $E_{t}^{p}$, can be calculated based on the power consumption of demand appliances as follows:

(3) $$E_{t}^{p}=E^{p}-\sum_{i=1}^{N}P_{a_{i}}\times \gamma_{a_{i}}^{t}.$$

Thereby, the objective function of the problem can be calculated as follows:

(4) $$\begin{array}{c} \sum_{i=1}^{N}E_{a_{i},t}^{c}\times \gamma_{a_{i}}^{t}\leq L_{t}+E_{t}^{p},\forall t\in T,\\ s.t.\;\gamma_{a_{i}}^{t}\in \left\{0,1\right\},\;\forall t\in T,\forall i\in N,\\ E_{t}^{p}\geq 0,\;\forall t\in T,\;\; \end{array}$$

where $L_{t}$ denotes the power limit for time slot *t*.

### 4.2. Rule-Based Smart Home Energy System

In this paper, the proposed approach for smart HEMS involves dynamic scheduling appliances in terms of automated and adaptive systems with the changes in environment (i.e., the power demand from non-scheduled appliances or the supply power from renewable resources) to meet the load balancing problem. Additionally, the scheduling process does not rely on any manual control or any centralized faculties. Specifically, with the advanced connected object technologies [26], each connected appliance can be regarded as an intelligent agent that can make individual decisions by communicating and collaborating with each other. In this regard, we consider a set of constraints for smart HEMS, which can be regarded as the negotiation protocol (knowledge based K) for connected appliances as follows:

- Total power consumption of appliances at each time slot should be limited to the allotted load capacity (Equation (4)).
- When the supply power is available, it should be prioritized for assigning to the appliances.
- The non-scheduled appliance would not be delayed and the status always switches ON when they send a request message.
- In the case of scheduled appliances, the one in a higher priority level is prioritized to use the resource.

Typically, the priority level among appliances in real time is an important factor for dynamic scheduling of connected appliances in terms of the satisfaction of customer. Basically, each scheduled appliance is set up with the start time $\Phi^{s}$, end time $\Phi^{e}$ and operation time $\Phi^{o}$. In this regard, the appliance should complete their operation before the end time $\Phi^{e}$. Hence, in this paper, the least slack time with rate (LSTR), which is a modified algorithm of least slack time (LST), is taken into account. Formally, the slack time $\Phi^{slack}$ for a task is calculated as follows:

(5) $$\Phi^{slack}=\left(\Phi^{e}-\Phi^{t}\right)-\Phi^{o^{\prime }},$$

where $\Phi^{t}$ and $\Phi^{o^{\prime }}$ are the real time since the task start and the remaining computation time, respectively. However, the original LST algorithm is not able to handle the idle state problem in real time [21,27]. Thus, the rate R between remaining time and execution time was proposed for determining the priority among appliances, which is defined as follows:

(6) $$R_{a_{i}}^{t}=\frac{\Phi_{a_{i}}^{o^{\prime }}}{\Phi_{a_{i}}^{e}-\Phi_{a_{i}}^{t}}.$$

### 4.3. Consensual Negotiation Model for Decentralized Decision-Making among Connected Appliances

The basis ideal of the decentralized approach for smart HEMS is that the connected appliances, based on the communication and collaboration with others, make decisions by themselves without any centralized facilities (e.g., HEMS controller). In this regard, the connected appliance needs to negotiate with other appliances in order to decide the proper scheduling following the rule-based system (Section 4.2). In order to deal with this problem, we propose a consensual negotiation approach for smart decision-making among connected appliances, which emerge as a promising approach for managing inter-agent dependencies at the operating time. Hence, we present the formal definition of consensus negotiation as follows:

**Definition** **3** **(Consensual** **negotiation).** Consensual negotiation is a decision-making process for connected appliances that seek an agreement for operating action.

Accordingly, a connected appliance needs to get permission from others for operating. In this regard, we apply a distributed process synchronization theory to solve the problem in which connected appliances refer to multiple processes that are joined up at a certain point (energy store), in order to reach an agreement regarding a certain sequence of action [17].

**Figure 7:**
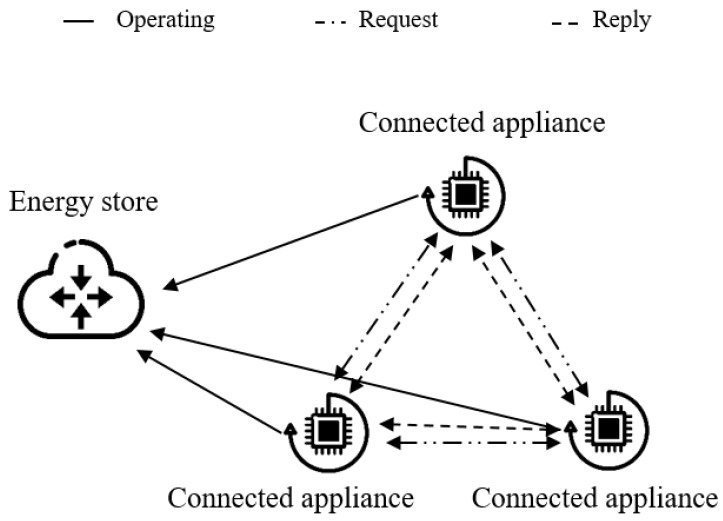
Consensual negotiation process for connected appliances.

Figure 7 depicts the negotiation process among connected appliances. Accordingly, we use the Inter-Process Communication (IPC) that includes Request and Reply messages. Moreover, there is a communication link between two processes (connected appliances) to communicate with each other by using Request and Reply functions. Specifically, the sequence of actions for an appliance can be defined as follows:

- Requesting for the operation: send Request(I,E).
- Executing the operation: receive Reply(I).
- Release after the operation: send Reply(I).

## 5. Consensual Negotiation-Based for Dynamic Load Balancing in Smart HEMS

This section presents the decentralized dynamic load balancing algorithm for connected appliances based on the consensual negotiation model in which connected appliances can make individual decisions following the rule-based smart energy management system. Particularly, the rough sketch of the proposed algorithm is given as follows:

(i) When a connected appliance $a_{i}$ requires an active operation, it sends a *Request Message* to all appliances that are operating and waits for their permission (Algorithm 1):
  - If appliance $a_{i}$ belongs to $T_{1}$, it changes state ($\gamma_{a_{i}}^{t}$:= On) and notifies all other appliances.
  - If appliance $a_{i}$ belongs to $T_{2}$, it will send a request message which includes the operation time ($\Phi^{o}$), the deadline ($\Phi^{e}$), and waiting for the reply message from others ($\gamma_{a_{i}}^{t}$:= Waiting).
  - If appliance $a_{i}$ belongs to $T_{3}$, surplus power ($E^{p}$) will be computed.
(ii) When connected appliance $a_{j}$ receives a request message from $a_{i}$ (Algorithm 2), it first checks the condition following Equation (4):
  - In the case that the current power consumption is lower than the allotted load capacity ($\left(\sum_{i=1}^{N}E_{a_{i},t}^{c}\times \gamma_{a_{i}}^{t}\right)\leq L_{t}-E_{t}^{p}$):
    - If the request message from appliance $a_{i}$ belongs to $T_{1}$ or $T_{3}$, the appliance $a_{j}$ only needs to update the information as their knowledge base (K) of the environment.
    - On the other hand, if the request message from the appliance is $T_{2}$, the priority level of two appliances will be taken into consideration (Equation (6)). Specifically, the appliance $a_{i}$ should send a Reply message if the priority rate of the appliance which sends the request is larger than $a_{i}$. Otherwise, the request message will be put in the pending list $P_{a_{i}}$ of appliance $a_{i}$.
  - In the case that current power consumption is lower than the allotted load capacity $(\left(\sum_{i=1}^{N}E_{a_{i},t}^{c}\times \gamma_{a_{i}}^{t}\right)>L_{t}-E_{t}^{p}$), some appliances in the flexible load shift model, which are operating, can be switched to *Inactive* based on priority level to maintain the load balancing.
(iii) As a result, an appliance, which belongs to $T_{2}$, can switch on when it receives all reply messages from others. In addition, when the appliances complete their operation, they should send a reply message to all the request messages in their pending list (Algorithm 3).

**Algorithm 1:** Request message of connected appliances
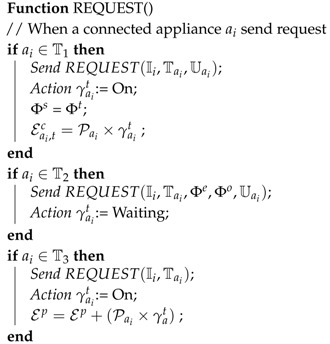

**Algorithm 2:** Reply message of connected appliances
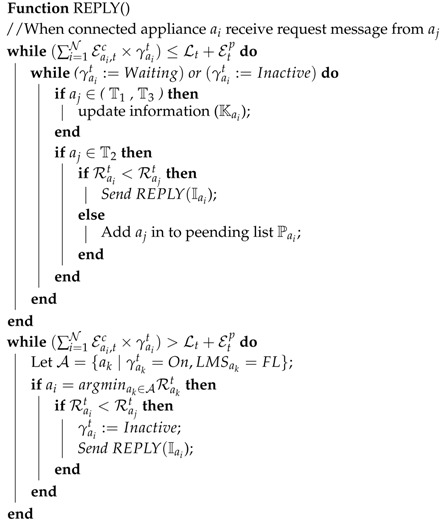

**Algorithm 3:** Release message of connected appliances
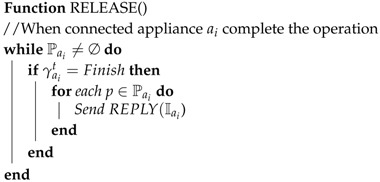



## 6. Simulation and Discussion

### 6.1. Developing a Smart HEMS Environment

To evaluate our approach, in this study, we develop a smart home system using *Netlogo simulator*, an Agent-Based Modeling environment, which follows the work in [28]. The simulation environment is written mostly in Scala, with some parts in Java and it works well with a PC with an CPU (Intel i7-4790 3.6 GHZ) and 16 GB main memory.

**Figure 8:**
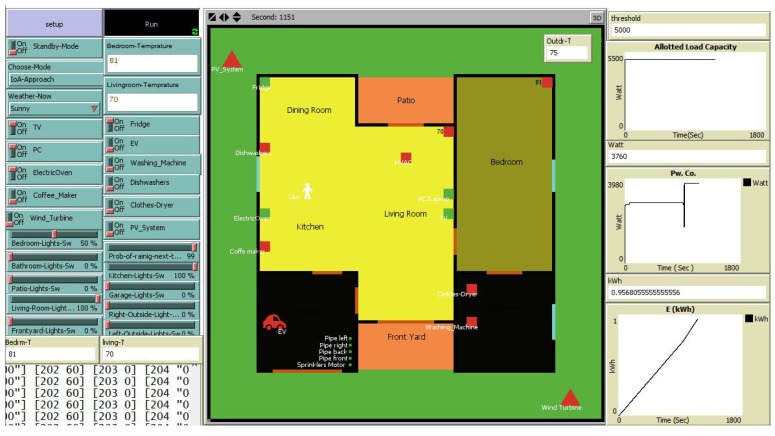
Simulated interface of smart HEMS.

In this regard, Figure 8 shows the interface of our smart home simulation in which the list of connected appliances and their properties for evaluating are shown in Table 1. Particularly, connected appliances of the smart home environment were tested during three hours in the morning (from 6:00 a.m. to 9:00 a.m.), which is the maximum time duration of customers using electric appliances when they wake up and start a new day. The request for operation (power demand) of connected appliances are randomly distributed based on their probabilities (Table 2). However, they should follow the context, for instance, smart light systems in each room will be switched on when users move in, and turned off when they leave, or the clothes dryer should be operated after the washing machine completes its operation, and so on. Moreover, we adopt a CPP tariff [29] for comparative analysis to show the effectiveness of our approach. Hence, we are able to determine the peak load and off-peak load of each time horizon T based on CPP signal (threshold load balancing values). The performance parameters are comprised of the load profile and the energy consumption, which are showed in the following subsection.

### 6.2. Numerical Results and Discussion

**Figure 9:**
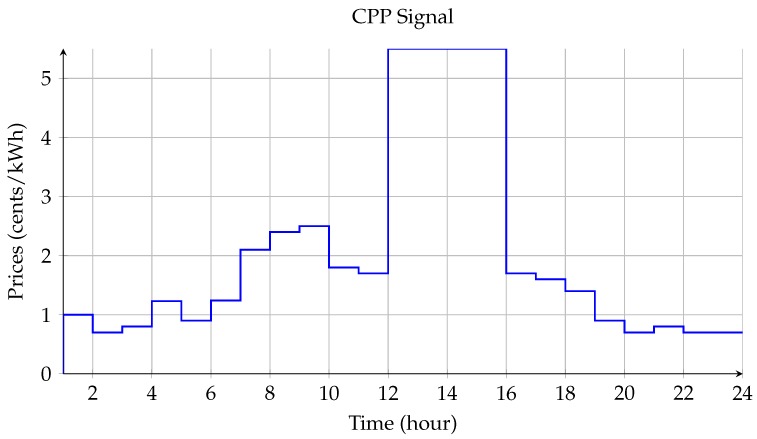
CPP tariff for each hour during a day.

As shown in Figure 9, the prices of using electric appliances increase from 6:00 a.m. to 9:00 a.m. In this regard, connected appliances tend to be scheduled between 6:00 a.m. and 7:00 a.m. for cost effectiveness. However, the total power consumption in each time slot should be limited following Equation (4).

**Figure 10:**
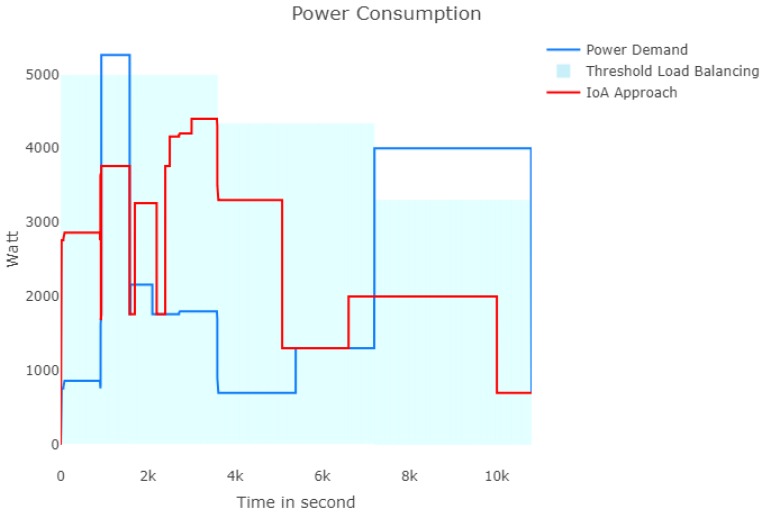
Power consumption per second.

**Figure 11:**
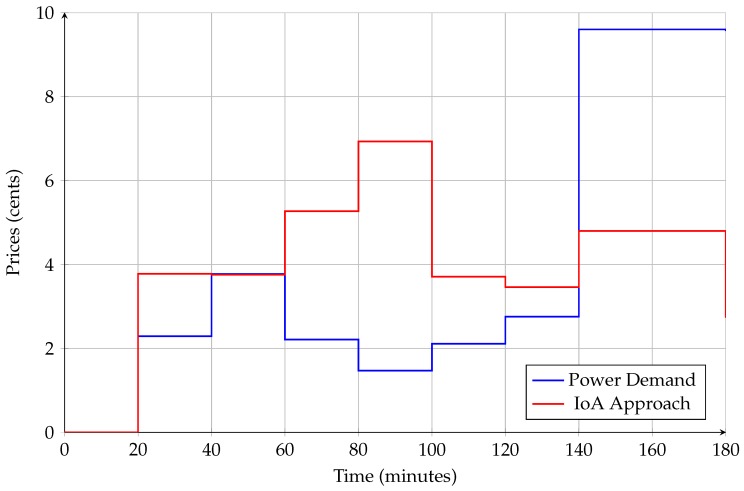
Cost consumption from 6:00 a.m. to 9:00 a.m.

Figure 10 depicts the load profile of our approach compared with the power demands of users. As result, the load balancing problem can be solved when the appliances communicate and collaborate with each other. As we mentioned above, the meaning of this result is that, by solving the load balancing problem in each time slot, the scheduled appliances can be self-adaptive so as to avoid the peak-load in order to improve the effectively of consumption cost. Particularly, the electric cost per time slot *t* can be calculated as follows:

(7) $$C_{t}=\sum_{i=1}^{N}E_{a_{i},t}^{c}\times \psi \left(t\right),$$

where ψ(t) indicates the electricity pricing tariff in time interval *t*. In this regard, the results in Figure 11 represent the electricity cost every 20 minutes from 6:00 a.m. to 9:00 a.m. of our approach. As a result, the total cost can be reduced by rescheduling the connected appliances from peak load to off-peak load. Particularly, the cost saving that can be reduced by applying the proposed approach is 4.144 cents (43.375 for power demand and 39.231 for the proposed approach).

## 7. Conclusions

A dramatic increase of domestic energy demand has motivated experts to implement energy efficient domestic appliances and environments. With the development of IoT, where IoT devices are able to connect and communicate with each other, many distributed approaches using intelligent technologies have been proposed for improving the performance of smart applications. Although there are still many challenges that need to be considered such as transmission, security, and delay messages problems of network connectivity, these smart scenarios will come out in the very near future. In this study, based on the advanced technologies of IoT, we present a new approach for smart HEMS. In this regard, connected appliances are able to be self-adaptive by communicating and collaborating for improving the performance of the system in terms of automatic and adaptiveness. Specifically, the IoA concept, which includes connected agents, has been proposed as a promising framework for different types of connected appliances. Thereby, connected appliances are able to make individual decisions for solving the load balancing problem based on a consensus algorithm. Regarding the experiment, we develop a smart home environment using Netlogo for evaluating the effectiveness of the proposed approach. In particular, we implement the system which includes 13 connected appliances during three hours in the morning, and the simulation came out with promising results. Extending the running time (e.g., one day, one week and so on) with the size of devices is our future direction for this study. Moreover, analyzing the context of users (e.g., user habits) to make smart decisions for scheduling appliances in terms of automatic and adaptive systems also needs to be taken into account.

## Figures and Tables

**Table 1:** List of Internet of Things (IoT)-based connected appliances.

Appliance List				
No.	Appliance Name	Type of Appliances	Load Shift Model	Power Rating (KW)
1	Fridge/freezer	$T_{1}$	NA	0.7
2	Lighting	$T_{1}$	NA	0.4
3	TV	$T_{1}$	NA	0.3
4	PC/laptop	$T_{1}$	NA	0.5
5	Coffee Maker	$T_{1}$	NA	1.5
6	Washing machine	$T_{2}$	FL	0.6
7	Dishwasher	$T_{2}$	GL	0.4
8	Clothes dryer	$T_{2}$	FL	1.3
9	Electric oven	$T_{2}$	GL	2
10	HVAC	$T_{2}$	FL	1
11	Electric vehicle	$T_{2}$, $T_{3}$	FL	2
12	PV system	$T_{3}$	NA	1.25
13	Wind turbine	$T_{3}$	NA	0.5

**Table 2:** Parameters of appliances [25].

No.	Appliance Name	Type	Time Operation (min)	Probability
1	Fridge/freezer	$T_{1}$	Always on	100%
2	Lighting	$T_{1}$	Depend on consumers	100%
3	TV	$T_{1}$	40	100%
4	PC/laptop	$T_{1}$	40	95%
5	Coffee Maker	$T_{1}$	8	53%
6	Washing machine	$T_{2}$	60	86%
7	Dishwasher	$T_{2}$	8	34%
8	Clothes dryer	$T_{2}$	60	8%
9	Electric oven	$T_{2}$	8	53%
10	HVAC	$T_{2}$	Depend on consumers	31%
11	Electric vehicle	$T_{2}$, $T_{3}$	60	50%
12	PV system	$T_{3}$	NA	10%
13	Wind turbine	$T_{3}$	NA	10%
